# Self-reported health among immigrants in Luxembourg: insights from a nationally representative sample

**DOI:** 10.1007/s10389-021-01648-1

**Published:** 2021-09-20

**Authors:** Launick Saint-Fort, Erik J. Rodriquez, Eliseo J. Pérez-Stable, Joël Billieux

**Affiliations:** 1 Department of Behavioural and Cognitive Sciences, University of Luxembourg, Esch-sur-Alzette, Luxembourg; 2 Department of Surgery, Division of Trauma and Acute Care Surgery, Penn Medicine Lancaster General Health,, Lancaster, PA, USA; 3 Division of Intramural Research, National Heart, Lung, and Blood Institute, Bethesda, MD, USA; 4 Office of the Director, National Institute on Minority Health and Health Disparities, Bethesda, MD, USA; 5 Institute of Psychology, University of Lausanne, Lausanne, Switzerland

**Keywords:** Immigrants, Population health, Health disparities, Self-reported health, Luxembourg

## Abstract

**Aim:**

Although immigrants account for nearly half of Luxembourg’s population, few studies have investigated differences in self-reported health by nationality in Luxembourg. Our study aimed to explore the association between nationality and self-reported health in Luxembourg.

**Subject and methods:**

Cross-sectional data from the 2015–2016 Panel Socio-Economique Liewen zu Lëtzebuerg (PSELL3) were used. Nationalities included Luxembourger, Portuguese, French, Italian, Belgian and German. Multivariable logistic regression analyses examined the association between nationality and three self-reported health measures: general health status, limitation in activity due to a health problem, and living with a chronic illness or condition.

**Results:**

Of 8084 participants, 65% were Luxembourgers, 20% were Portuguese, and the remaining 15% were French, Italian, Belgian, or German. Italian nationals were more likely to report fair, poor, or very poor health [aOR = 1.54; 95% CI = 1.07, 2.22] and Portuguese nationals demonstrated both higher odds of fair, poor, or very poor health [aOR = 1.57; 95% CI = 1.28, 1.92] and limitation in activity [aOR = 1.32; 95% CI = 1.07, 1.64] compared to Luxembourgers. However, Portuguese nationals were also less likely to report living with a chronic illness [aOR = 0.79; 95% CI = 0.63, 0.98]. In education-stratified models, primary-educated Portuguese nationals were more likely to report fair, poor, or very poor health [aOR = 1.78, 95% CI = 1.36, 1.92] and limitation in activity [aOR = 1.36, 95% CI = 1.04, 1.79], but not less likely to report living with a chronic illness.

**Conclusions:**

Nationality and education level should be considered in future studies concerning self-reported health in Luxembourg. Further research is needed to examine disparities in self-reported health among Portuguese and Italian nationals.

## Introduction

Although the Grand-Duchy of Luxembourg has the fourth highest life expectancy at birth (82.1 years of age) in Europe, chronic conditions remain as the leading contributors to poor health and mortality in Luxembourg (OECD 2019a). A distinct characteristic of the Grand-Duchy is its large immigrant population, resulting from the rise of the steel industry in the mid-nineteenth century (Kollwelter 2007). Luxembourg’s thriving economy, and its being a hub for financing services, are the primary motives for foreign nationals to reside in the country (Gouvernement du Grand-Duché du Luxembourg 2021b). Other contributing factors include the following: a multilingual environment, health insurance and social security systems, high quality infrastructure, and governmental support for families (Gouvernement du Grand-Duché du Luxembourg 2020). Today, immigrants account for nearly half of Luxembourg’s population, with the Portuguese (15.6%) comprising its largest international community followed by the French (7.6%), Italians (3.7%), Belgians (3.3%), and Germans (2.1%) (Gouvernement du Grand-Duché du Luxembourg 2021a). Despite the nation’s increasing immigrant population, mainly citizens of other European Union (EU) nations, no study to date has investigated the heterogeneity in self-reported health by nationality in Luxembourg.

Self-reported health has been found to be a strong predictor of mortality (Menec et al. 1999), morbidity (Emmelin et al. 2003; Latham and Peek 2013), and health service utilization (George et al. 2012) outcomes. Previous studies on immigrant health have shown that factors associated with migration — such as transitioning to a new environment, social isolation, ethnic discrimination, and job instability — can influence health outcomes and integration into the host country (Phinney et al. 2001; Hernández-plaza et al. 2004). Due to recent changes in the economic and social landscape within and around the continent, immigration throughout Europe has increased significantly (de Haas et al. 2018). This has prompted researchers to begin exploring health outcomes among immigrants in numerous European nations. Studies on immigrant health in Europe, though scarce and at times with findings which are contradictory, have shown that immigrant groups differ in health status. For example, immigrants in Spain (Hernández-Quevedo and Jiménez-Rubio 2009) reported better health status than their native counterparts. Additionally, immigrants in France (Dourgnon et al. 2008) and the UK (Chandola 2001) reported poorer health status. Health status can also vary among European nationals immigrating to other European nations. Findings on the self-reported health of immigrants in Switzerland showed that self-reported health of German and French immigrants were comparable to that of the majority Swiss population. This compares to immigrants from Italy, Portugal, and Spain, who reported lower levels of health in several areas (Bischoff and Wanner 2008).

In Luxembourg, few studies have investigated variations in measures of health among immigrants. A study evaluating depression in Luxembourg found that second-generation immigrants had a higher odds of having depressive symptoms than non-immigrants, regardless of socioeconomic status and behavioral characteristics such as alcohol consumption and physical activity (Ruiz-Castell et al. 2017). However, the study did not account for nationality, even though the prevalence of depression can differ by nationality in Europe (Bermejo et al. 2012; Toselli et al. 2014). Another study assessing risk factors, which contribute to overweight and obesity statistics in Luxembourg, revealed that men born in a non-European country and women born in Portugal were more likely to be overweight (Samouda et al. 2018). Furthermore, immigrant groups were not disaggregated by nationality except for Portuguese.

Considering that EU nationals constitute the largest immigrant population in Luxembourg, examining the association between nationality and measures of health will provide a clearer understanding of the health profile of Luxembourg’s population. To expand the literature on the health status of the overall population of Luxembourg, our study evaluated self-reported health among six European nationalities residing in the country. Particularly, the associations between nationality and self-reported general health status, limitation in activity due to a health problem, and living with a chronic illness or condition were evaluated.

## Subject and methods

### Data collection

Cross-sectional, individual-level data employed in this study came from the 2015–2016 Panel Socio-Economique Liewen zu Lëtzebuerg (PSELL3 “© ORIGINE LISER: RIGHTS RESERVED TO LISER 2017”). PSELL3 employed a multi-stage design where households are first sampled, followed by interviews of civilian, non-institutionalized, household members, aged 16 or older, to assess and evaluate living conditions of the population residing in the Grand-Duchy of Luxembourg. A civilian, non-institutionalized, population is defined as those who are not inmates of penal institutions, residents of mental health facilities or nursing homes, or on active duty in the Armed Forces. The panel was initiated in 2003, and measures social and economic indicators including some measures of health (EU-SILC / PSELL 2014). PSELL3 incorporated both cross-sectional and longitudinal data collected by the Luxembourg Institute of Socio-Economic Research (LISER), and was designed using guidelines and methodologies implemented by the European Union Statistics on Income and Living Conditions (Eurostat). Weighting factors included probability of selection and nonresponse, and were adjusted to the distribution of households and target population by sex, age (5-year age groups), household size/composition, and region. In total, 8767 people were surveyed in the 2015–2016 panel. As publicly available data from PSELL3 are secondary and de-identified, this study was deemed exempt from institutional review board (IRB) approval as per the U.S. Code of Federal Regulations, Title 45, Part 46.

### Participants

Participants included in this analysis were those who self-identified as one of the following nationalities: Luxembourger, Portuguese, French, Italian, Belgian, and German (*n* = 8084). The sample was restricted to the aforementioned nationalities due to their sizeable population proportions compared to other EU and non-EU foreign nationalities in Luxembourg (2019). A total of 683 participants who did not meet the nationality criteria of this study were excluded from the analyses.

### Measures

#### Control variables

Age in years, gender (men, women), marital status (never married, married, separated, divorced, widowed, and partnered), and employment status (employed, unemployed, retired, and other [not specified]) were assessed by self-report. Educational attainment was categorized as primary (lower secondary or less), secondary (upper secondary), and tertiary (post-secondary: trade school, college, or university graduate). Frequency of getting together with family and frequency of getting together with friends were included to assess social connections. Response options for both variables were recategorized as > once a month (daily, every week, several times a month) or ≤ once a month (once a month, at least once a year, never). Additional variables that were considered, but were found to have approximately 50% missingness, were duration of stay (in years) and type of labor: blue collar (manual), white collar (non-manual) and other (family caregiver, self-employed). For type of labor, missingness was consistently around, or greater than, 50% for each nationality included in this study.

#### Self-reported health

Three measures of health were assessed in this study. First, self-reported general health status, inquired as “how would you describe your health in general?”, was dichotomized as “very good or good” if participants reported “very good” or “good” health status and “fair, poor, or very poor” for those who reported “fair”, “poor”, or “very poor” health status. Second, limitation in activity due to a health problem was defined as “for at least the past 6 months, to what extent have you been limited because of a health problem in activities people usually do?” Participants who responded, “limited but not severely limited” or “severely limited” in activity were categorized as “yes” and those who responded “not limited at all” were categorized as “no”. Third, participants were asked “do you have any chronic (long-standing) illness or condition?”, for which they responded “yes” or “no”. The survey did not provide any additional instructions to participants framing any of these questions.

### Analysis

PSELL3 survey weights were applied to allow for nationally representative sociodemographic statistics and multivariable logistic regression estimates. Means, standard errors, one-way ANOVA, and Tukey post-hoc tests were calculated for age and duration of stay. Frequencies, chi-square, and adjusted Pearson residual post-hoc tests were conducted for gender, educational attainment, marital status, employment status, type of labor, frequency of getting together with family, and frequency of getting together with friends. Multivariable logistic regression models were used to examine the association between nationality and the three measures of health: self-reported general health status, limitation in activity due to a health problem, and living with a chronic illness or condition. Due to the large proportion of Portuguese nationals with a primary level of education, additional multivariable logistic regression models were generated, and stratified by educational attainment, to further explore the link between nationality and measures of health. All models were adjusted for age, gender, educational attainment, marital status, employment status, and frequencies of getting together with family and friends. It is important to note that social connection variables employed in the multivariable logistic regression analyses were not dichotomized. This was done to prevent any possible reductions in variance to be explained by these two variables. Duration of stay and type of labor were not included in the multivariable logistic regression models due to their extensive missingness and the possibility of them introducing bias into the study because of such missingness. All analyses included participants with non-missing values for all variables, and were conducted utilizing Stata/IC, Version 15 (Stata Corp LP, College Station, TX, USA).

## Results

### Sociodemographic characteristics and self-reported health

Sociodemographic characteristics of participants included in this study are shown in Table 1. Approximately 65% of the sample were Luxembourger, with Portuguese being the largest EU foreign nationality (20.2%) followed by French (6.4%). Age differed significantly between nationalities as determined by one-way ANOVA (*F* (5, *N* = 8078) = 40.8, *p* < 0.001). A Tukey post-hoc assessment revealed that Portuguese (34.3 ± 19.0 years) and French (36.0 ± 20.0 years) participants were significantly younger (−6.78 ± 0.61, *p* < 0.001 and − 4.99 ± 0.99, *p* < 0.001 respectively) than Luxembourgers (41.0 ± 22.6 years), whereas Italians (47.8 ± 21.5 years) and Germans (48.0 ± 18.5 years) were significantly older (6.73 ± 1.33, *p* < 0.001 and 7.05 ± 1.82, *p* < 0.001 respectively) than Luxembourgers. There was no statistically significant difference in age between Belgians and Luxembourgers.

Most respondents by nationality were men, with the exception of French (51.8% women) and German (54.0% women). Level of education differed significantly by nationality (*X*^2^(10, *N* = 6590) = 1.1e + 03, *p* < 0.001). Across all foreign nationalities, Belgian and French participants reported higher proportions of tertiary educational attainment (56.2% and 58.5% respectively) than Luxembourgers (26.7%). In comparison, only 4.3% of Portuguese participants reported having attained a tertiary level of education. Adjusted Pearson residuals indicated that (a) primary education and Portuguese nationality were observed significantly more than expected, and (b) French nationality with primary education were observed significantly less than expected.

The proportions of all measures of health differed significantly by nationality (general health status [*X*^2^(5, *N* = 6635) = 60.6, *p* < 0.001], limitation in activity [*X*^2^(5, *N* = 6636) = 28.7, *p* < 0.001] and living with a chronic illness [*X*^2^(5, *N* = 6633) = 30.2, *p* < 0.001]). Greater than 35% of Portuguese and 40.6% of Italians reported having fair, poor, or very poor health, which were the highest among all nationalities (Table 1). French (76.1%), Belgian (76.1%), and Luxembourgers (70.4%) reported the highest percentages of very good or good health status. German participants reported the highest percentage of limitation in activity due to a health problem (33.5%) as well as living with a chronic illness (31.2%). However, French participants reported the lowest percentage for both measures of health (15.6% and 18.0% respectively). Just over 25% of Luxembourgers reported living with a chronic illness, and 26.4% experienced limitation in activity due to a health problem. Adjusted Pearson residuals revealed that fair, poor, or very poor health was observed significantly more than expected among Portuguese and Italian nationals. Results were observed significantly less than expected among French nationals with respect to reported limitation in activity due to a health problem, and among both French and Portuguese living with a chronic illness. No significance between the observed and expected values of limitation in activity were apparent among Portuguese nationals.

### Multivariable analyses of associations between nationality and measures of health

Associations between nationality and measures of health are displayed in Table 2. In the overall sample, the multivariable model predicting fair, poor, or very poor health status, adjusted for sociodemographic characteristics, showed that Portuguese and Italian nationals were more likely to report fair, poor, or very poor health [aOR = 1.57; 95% CI = 1.28, 1.92 and aOR = 1.54; 95% CI = 1.07, 2.22 respectively] compared to their Luxembourger counterparts. Lower odds of limitation in activity [aOR = 0.66; 95% CI = 0.45, 0.96] were observed among French nationals, and higher odds of limitation in activity [aOR = 1.32; 95% CI = 1.07, 1.64] were seen among the Portuguese. Portuguese nationals were also less likely [aOR = 0.79; 95% CI = 0.63, 0.98] to report living with a chronic illness compared to Luxembourgers.

Results from Table 2 show that age was statistically significant across all measures of health and the results were as follows: fair, poor, or very poor health status [aOR = 1.04, 95% CI = 1.03, 1.05], limitation in activity [aOR = 1.03, 95% CI = 1.03, 1.04], and living with a chronic illness [aOR = 1.02, 95% CI = 1.01, 1.03]. Women were more likely than men to report living with a chronic illness [aOR = 1.25; 95% CI = 1.07, 1.46] and having limitation in activity [aOR = 1.43; 95% CI = 1.22, 1.67] (Table 2). Compared to a tertiary level of education, both primary [aOR = 1.98, 95% CI = 1.59, 2.48] and secondary [aOR = 1.57, 95% CI = 1.27, 1.94] levels of education increased the odds of fair, poor, or very poor health. Education level was also significantly associated with limitation in activity [primary: aOR = 1.78, 95% CI = 1.42, 2.24; secondary: aOR = 1.55, 95% CI = 1.24, 1.93] and living with a chronic illness [primary: aOR = 1.76, 95% CI = 1.41, 2.20; secondary: aOR = 1.53, 95% CI = 1.24, 1.89]. For every 1-unit increase in frequency in getting together with family, lower odds of reporting fair, poor, or very poor health [aOR = 0.88, 95% CI = 0.83, 0.93], limitation in activity [aOR = 0.92, 95% CI = 0.88, 0.98], and living with a chronic illness [aOR = 0.92, 95% CI = 0.87, 0.97] were observed. Greater frequency of getting together with friends was only protective for reporting fair, poor, or very poor health [aOR = 0.87, 95% CI = 0.82, 0.92].

When stratified by educational attainment (Table 3), Portuguese nationals with a primary level of education were more likely to report fair, poor, or very poor health status [aOR = 1.78, 95% CI = 1.36, 1.92] and limitation in activity [aOR = 1.36, 95% CI = 1.04, 1.79] compared to Luxembourg nationals with a primary education. In contrast, French nationals with a primary level of education were less likely [aOR = 0.29, 95% CI = 0.13, 0.68] to report limitation in activity. There were no statistically significant differences in living with a chronic illness among any nationalities with a primary education, which differ from results among Luxembourgers in the non-stratified models. Across all nationalities with greater than a primary level of education, point estimates of the three measures of health status trended towards those observed in the non-stratified models, but none were statistically significant.

## Discussion

This study investigated relationships between nationality and self-reported health among residents of Luxembourg, using data from the nationally representative PSELL3 collected in 2015–2016. Findings from overall models suggested that Portuguese and Italian participants were significantly more likely to experience fair, poor, or very poor health. Portuguese participants were also more likely to encounter limitation in activity due to a health problem and less likely to be living with a chronic illness compared to Luxembourgers. When stratified by educational attainment, only Portuguese with a primary level of education were more likely to report fair, poor, or very poor health and limitation in activity. Additionally, they were not less likely to report chronic illness. The mixed findings among Portuguese nationals in our overall models seemed to be partially clarified by results from the education-stratified results, where only Portuguese nationals with a primary level of education were at greater odds of worse self-reported health than Luxembourgers.

### General health status

The increased likelihood of fair, poor, or very poor self-reported health status observed among Portuguese and Italian nationals in our overall models, even after adjusting for educational attainment, is notable. Several studies have shown an association between education and health status, where individuals with higher education are more likely to report good health than those with less formal years of education (Mackenbach et al. 2008; Mackenbach 2012). In our study, over 75% of Portuguese nationals, followed by 44% of Italians, reported having only a primary education. In comparison, other nationalities such as the French, Germans, and Belgians were among the highest educated, even more so than Luxembourgers. When we stratified by educational attainment, Portuguese with a primary education were more likely to self-report fair, poor, or very poor health. Given the large disparity in educational attainment among Portuguese and Italian nationals, as well as the results of our stratified models, it is likely that the higher odds of having fair, poor, or very poor health is influenced by their generally lower socioeconomic status, particularly among the Portuguese.

Furthermore, differences in perceived health status by nationality may be influenced by social and cultural factors (Agyemang et al. 2006; Wijekoon Mudiyanselage et al. 2018). For example, although life expectancy in Portugal (81.6 years) is slightly higher than the EU average (80.9 years), the Portuguese report poorer health than their EU counterparts. In 2017, less than half of the population in Portugal reported being in good health, compared to more than two-thirds of adults in the EU overall (OECD 2019c). Nearly 80% of the population in Italy reported being in good health, and inequalities in life expectancy are less prominent than in other EU countries (OECD 2019b). Yet in our study, Italians were more likely than Luxembourgers to report fair, poor, or very poor health, indicating potential differences in cultural interpretation of health status among Italians living in Luxembourg compared to Italians living in Italy. Consequently, the apparent independent effect of Portuguese and Italian nationality, in Luxembourg, may merit further research to reveal the underlying factors contributing to poorer health status among these groups.

It is unsurprising that statistically significant differences in health status were not observed among French, Belgian, and German nationals, given that their gross domestic product (GDP) are similar. GDP is the most important indicator to capture economic activity and according to current data, the GDP of France, Belgium, Germany, and Luxembourg are all above the EU average, whereas the GDP of both Portugal and Italy are below the EU average (OECD 2021). It is plausible that the socioeconomic status of immigrants migrating from these neighboring countries may be more comparable to that of Luxembourg as opposed to immigrants from Portugal and Italy. This is further evident by similarities in the rates of good health status between Luxembourg, France, Germany, and Belgium (OECD 2019a), which are generally impacted by socioeconomic status. Thus, the lack of significance in outcomes among these groups are probably due to similar socioeconomic conditions among Luxembourg and its neighbors.

Due to the large proportion of primary educational attainment among Portuguese nationals, models were stratified by education level to determine if the relationship between Portuguese nationality and self-reported health outcomes were driven by lower educational attainment. None of the other nationality groups had significantly increased fair, poor, or very poor self-reported health among those with primary education, even though Italians had an adjusted odds ratio of 1.42. Furthermore, differences by nationality disappeared among those with greater than primary education.

### Limitation in activity due to a health problem

The increased likelihood of having limitation in activity due to a health problem among Portuguese nationals overall and among primary-educated Portuguese specifically may possibly be attributed to occupational factors, which are known to influence differences in activity limitations (Emslie et al. 1999; Messing and Silverstein 2009). Previous research has suggested that heavy physical work load and prolonged standing at work (Waters and Dick 2015) are associated with higher probabilities of physical functional limitations (Chau et al. 2009), absences because of illness (Voss et al. 2004), and work-related disability (Solovieva et al. 2018).

In Luxembourg, work in construction is predominantly occupied by Portuguese nationals (Gouvernement du Grand-Duché du Luxembourg 2015). As a result, the overrepresentation of Portuguese nationals in a physically demanding sector, and the conditions associated with such an occupation, may have resulted in adverse health over time and thereby could be related to higher odds of limitation in activity among this group. These outcomes reflect European data which showed that when comparing the countries of origin of the nationalities included in our study, the prevalence of self-reported long-standing limitations due to health problems was highest in Portugal. The prevalence of long-standing limitations was lower in Italy, Germany, and France and higher in Belgium compared to Luxembourg (Eurostat 2020).

### Living with a chronic illness

Despite higher odds of fair, poor, or very poor health and limitation in activity, Portuguese nationals in Luxembourg were less likely to report living with a chronic illness, even after age-adjustment, in non-stratified models. These contrasting results were unexpected, given that previous studies have shown an association between self-reported general health status and chronic illness (Wu et al. 2013; Van der Heyden et al. 2014). Our findings among the Portuguese suggested potential underlying issues with measuring chronic illness. Given the subjective nature of self-reported health items, we cannot exclude the possibility of discrepancies between participant responses and objective diagnoses by physicians.

Participant self-reporting of diagnoses presumes that the respondent possesses the necessary knowledge to accurately report on chronic illnesses and that the questionnaire is sufficient in facilitating the inquiry. Studies assessing agreement between health administrative data and self-reported chronic conditions suggest that agreement varies according to the chronic condition being measured. For diseases that are unambiguous, such as diabetes, agreement was higher than poorly defined diseases such as congestive heart failure (Tu et al. 2007; Singh 2009). Participant responses may also be impacted by other factors. For instance, the participant may be asymptomatic, is not limited by their condition, or may not fully comprehend their diagnosis (Simpson et al. 2004). Moreover, individuals with lower education levels were found to demonstrate lower health literacy skills than those with higher educational attainment (Berkman et al. 2011). Because health literacy impacts use of healthcare services (Lee et al. 2010) and the ability to report whether one has been diagnosed with a chronic illness (Toci et al. 2015), level of education could also play a role. Statistics on chronic diseases in Europe revealed that chronic illnesses were more common in Germany and Portugal, and less common in France, Italy, and Belgium, compared to Luxembourg (OECD 2019”).

When stratified by education, the lower odds of reporting living with a chronic illness among primary educated Portuguese nationals was no longer significant. These results suggest that lower health literacy among Portuguese nationals, due to the predominance of primary level of education, was perhaps not the primary driver of the reduced odds of reported chronic illness. It is also possible that in a future study, with larger sample sizes, statistical significance may be observed among nationalities in education-stratified models. Although measurement issues may still exist among those with lower education levels, future research is needed to assess whether health literacy has an impact on self-reported health outcomes among Portuguese nationals.

### Study limitations

This study had several limitations. First, the cross-sectional nature of the study did not allow us to establish temporality. Second, relatively small sample sizes among some nationalities limited statistical power to detect differences. This may be particularly relevant for Portuguese and Italian nationals with a primary level of education and its influence on wider confidence intervals. Furthermore, because Luxembourg does not allow the collection of data regarding race and ethnicity, we were unable to account for possible racial or ethnic differences within and between nationalities, although the overwhelming majority would likely be categorized as White in the USA. Living with a chronic illness, as an item, was not exhaustive and therefore may have hindered our ability to determine whether participants understood which medical conditions were classified as chronic illnesses. We were also unable to consider generational status, since this item was not available in the PSELL3 dataset as well as duration of stay and type of labor due to these variables having approximately 50% missing data. Lastly, generalizability may not extend to immigrant populations whose nationalities were not surveyed in this study.

## Conclusion

Understanding differences in self-reported health of immigrants by nationality is important, as ethnic health disparities may be related to immigration and the ethnic identity of the country of origin. Self-reported health status was significant among Portuguese and Italian nationals in comparison to other immigrants and native Luxembourgers. Further studies concerning the health of Portuguese and Italian immigrants are needed to examine potential reasons for differences in self-reported health, including the relationship between socioeconomic status and health status.

## Figures and Tables

**Table 1:** Sociodemographic characteristics and measures of health by nationality: 2015–2016 Panel Socio-Economique Liewen zu Lëtzebuerg III (PSELL3): *n* = 8084

Characteristic	Nationality					
	Luxembourger *n*=5264	Portuguese *n*=1631	French *n*=521	Italian *n*=276	Belgian *n*=248	German *n*=144
Age, mean±SD*	41.0 (22.6)	34.3 (19.0)	36.0 (20.0)	47.8 (21.5)	43.0 (21.6)	48.0 (18.5)
Gender, %^a^						
Men	50.2	51.6	48.2	55.0	53.7	46.0
Women	49.8	48.4	51.8	45.0	46.3	54.0
Duration of stay (in years), mean±SD*	–	18.0 (12.6)	14.7 (11.1)	29.0 (19.4)	21.1 (13.7)	19.4 (14.8)
Educational attainment, %*^,a^						
Primary	32.8	75.5	12.9	44.4	11.6	17.9
Secondary	40.5	20.2	28.7	22.3	32.2	37.2
Tertiary	26.7	4.3	58.5	33.4	56.2	44.9
Marital status, %*^,a^						
Never married	44.0	46.0	49.7	35.6	36.4	30.0
Married	36.1	43.2	29.4	46.4	48.6	42.2
Separated	0.5	1.1	1.3	0.7	0.7	1.6
Widowed	6.2	2.0	1.1	9.9	3.9	4.7
Divorced	8.0	6.7	10.6	6.4	7.4	16.2
Partnered	5.3	1.0	8.0	1.2	3.1	5.3
Employment status, %*^,a^						
Employed	42.0	50.8	53.8	43.7	51.0	42.5
Unemployed	2.0	6.1	5.2	4.0	3.7	3.4
Retired	24.3	10.5	8.3	26.6	16.9	23.7
Other	31.7	32.6	32.7	25.6	28.3	30.4
Type of occupation, %*^,a^						
White collar	60.4	14.5	55.5	20.3	80.2	53.3
Blue collar	29.5	81.5	39.6	71.1	14.4	26.7
Other	10.1	4.1	4.9	8.6	5.4	19.9
General health status, %*^,a^						
Very good	23.3	18.5	32.5	17.4	32.3	29.5
Good	47.1	45.1	43.7	42.0	43.9	40.1
Fair	21.4	23.2	19.2	31.4	17.6	18.6
Poor	7.4	10.9	3.6	8.2	6.3	6.8
Very poor	0.8	2.2	1.1	1.1	0.0	5.0
Limitation in activity due to a health problem, %*^,a^						
No	73.6	70.9	84.4	67.6	76.9	66.5
Yes	26.4	29.2	15.6	32.4	23.2	33.5
Living with a chronic illness, %*^,a^						
No	74.6	79.7	82.1	74.0	77.3	68.9
Yes	25.5	20.3	18.0	26.0	22.7	31.2
Frequency of getting together with family, %*^,a^						
> once a month	78.0	63.0	42.5	62.8	57.5	37.1
≤ once a month	22.0	37.0	57.5	37.3	42.5	62.9
Frequency of getting together with friends, %^,a^						
> once a month	80.1	82.0	73.6	77.1	72.5	70.1
≤ once a month	19.9	18.0	26.4	22.9	27.5	29.4

*Note*. *SD* standard deviation

* *p* < 0.001 based on one-way ANOVA for continuous variables or chi-square test for categorical variables

a Weighted percentages may not sum to 100% due to rounding

**Table 2:** Weighted multivariable logistic regression results for six nationalities in Luxembourg, compared to Luxembourgers, and their odds of three measures of health: 2015–2016 Panel Socio-Economique Liewen zu Lëtzebuerg III (PSELL3)

Variables	Fair, poor, or very poor health status			Limitation in activity			Living with a chronic illness		
	aOR	95% CI	*P*	aOR	95% CI	*P*	aOR	95% CI	*P*
Main variable of interest									
Nationality									
Luxembourger		Reference			Reference			Reference	
Portuguese	**1.57**	**∣1.28, 1.92∣**	**< 0.001**	**1.32**	**∣1.07, 1.64∣**	**0.010**	**0.79**	**∣0.63, 0.98∣**	**0.035**
French	1.00	∣0.72, 1.40∣	0.980	**0.66**	**∣0.45, 0.96∣**	**0.028**	0.88	∣0.61, 1.27∣	0.499
Italian	**1.54**	**∣1.07, 2.22∣**	**0.020**	1.25	∣0.88, 1.77∣	0.209	0.90	∣0.62, 1.30∣	0.569
Belgian	0.83	∣0.53, 1.29∣	0.406	0.91	∣0.58, 1.43∣	0.675	0.93	∣0.61, 1.42∣	0.739
German	0.92	∣0.55, 1.51∣	0.735	1.29	∣0.78, 2.15∣	0.321	1.26	∣0.77, 2.08∣	0.362
Control variables									
Age	**1.04**	**∣1.03, 1.05∣**	**< 0.001**	**1.03**	**∣1.03, 1.04∣**	**< 0.001**	**1.02**	**∣1.01, 1.03∣**	**< 0.001**
Sex									
Men		Reference			Reference			Reference	
Women	1.10	∣0.95, 1.28∣	0.211	**1.43**	**∣1.22, 1.67∣**	**< 0.001**	**1.25**	**∣1.07, 1.46∣**	**0.005**
Educational attainment									
Tertiary		Reference			Reference			Reference	
Primary	**1.98**	**∣1.59, 2.48∣**	**< 0.001**	**1.78**	**∣1.42, 2.24∣**	**< 0.001**	**1.76**	**∣1.41, 2.20∣**	**< 0.001**
Secondary	**1.57**	**∣1.27, 1.94∣**	**< 0.001**	**1.55**	**∣1.24, 1.93∣**	**< 0.001**	**1.53**	**∣1.24, 1.89∣**	**< 0.001**
Marital status									
Never married		Reference			Reference			Reference	
Married	0.93	∣0.75, 1.16∣	0.521	1.00	∣0.80, 1.27∣	0.949	1.01	∣0.80, 1.27∣	0.904
Separated	1.71	∣0.85, 3.44∣	0.129	1.72	∣0.83, 3.55∣	0.142	1.94	∣0.92, 4.09∣	0.081
Widowed	0.85	∣0.60, 1.20∣	0.367	1.12	∣0.79, 1.60∣	0.521	1.16	∣0.81, 1.68∣	0.416
Divorced	1.15	∣0.86, 1.54∣	0.329	1.32	∣0.99, 1.78∣	0.059	1.26	∣0.94, 1.69∣	0.117
Partnered	1.31	∣0.86, 1.99∣	0.202	1.19	∣0.77, 1.83∣	0.419	1.33	∣0.88, 2.00∣	0.165
Employment status									
Employed		Reference			Reference			Reference	
Unemployed	**3.10**	**∣2.21, 4.36∣**	**< 0.001**	**2.86**	**∣2.02. 4.03∣**	**< 0.001**	**2.45**	**∣1.70, 3.53∣**	**< 0.001**
Retired	**1.49**	**∣1.18, 1.87∣**	**0.001**	**1.70**	**∣1.34, 2.15∣**	**< 0.001**	**2.00**	**∣1.58, 2.50∣**	**< 0.001**
Other	**1.33**	**∣1.05, 1.68∣**	**0.015**	**1.47**	**∣1.15, 1.87∣**	**0.002**	**1.29**	**∣1.03, 1.61∣**	**0.024**
Frequency of getting together with family	**0.88**	**∣0.83, 0.93∣**	**< 0.001**	**0.92**	**∣0.88, 0.98∣**	**0.011**	**0.92**	**∣0.87, 0.97∣**	**0.003**
Frequency of getting together with friends	**0.87**	**∣0.82, 0.92∣**	**< 0.001**	0.95	∣0.90, 1.01∣	0.105	0.95	∣0.89, 1.00∣	0.070

*Note*. *aOR* adjusted odd ratios, *CI* confidence interval, *P p* value.

Bold values indicate statistical significance.

**Table 3:** Weighted multivariable logistic regression results, stratified by educational attainment, for six nationalities in Luxembourg, compared to Luxembourgers, and their odds of three measures of health: 2015–2016 Panel Socio-Economique Liewen zu Lëtzebuerg III (PSELL3)

Main variable of interest	Fair, poor, or very poor health status			Limitation in activity			Living with a chronic illness		
	aOR	95% CI	*P*	aOR	95% CI	*P*	aOR	95% CI	*P*
Primary education only									
Nationality									
Luxembourger		Reference			Reference			Reference	
Portuguese	**1.78**	**∣1.36, 1.92∣**	**< 0.001**	**1.36**	**∣1.04, 1.79∣**	**0.024**	0.79	∣0.60, 1.06∣	0.116
French	0.65	∣0.33, 1.29∣	0.213	**0.29**	**∣0.13, 0.68∣**	**0.004**	0.70	∣0.27, 1.78∣	0.455
Italian	1.42	∣0.86, 2.32∣	0.167	1.25	∣0.88, 1.77∣	0.073	1.13	∣0.67, 1.90∣	0.631
Belgian	1.09	∣0.29, 4.10∣	0.899	1.10	∣0.28, 4.31∣	0.886	0.86	∣0.32, 2.27∣	0.756
German	0.62	∣0.26, 1.50∣	0.290	0.94	∣0.38, 2.36∣	0.900	1.15	∣0.44, 2.98∣	0.779
Greater than primary education									
Nationality									
Luxembourger		Reference			Reference			Reference	
Portuguese	1.43	∣0.99, 2.06∣	0.055	1.35	∣0.91, 2.01∣	0.139	0.81	∣0.54, 1.23∣	0.328
French	0.96	∣0.67, 1.36∣	0.800	0.70	∣0.48, 1.04∣	0.080	0.86	∣0.59, 1.42∣	0.462
Italian	1.56	∣0.95, 2.57∣	0.080	0.95	∣0.53, 1.68∣	0.851	0.67	∣0.37, 1.23∣	0.198
Belgian	0.73	∣0.45, 1.16∣	0.181	0.83	∣0.51, 1.33∣	0.437	0.89	∣0.55, 1.42∣	0.614
German	0.94	∣0.52, 1.68∣	0.831	1.32	∣0.74, 2.36∣	0.344	1.24	∣0.70, 2.20∣	0.469

*Note*. *aOR* adjusted odd ratios, *CI* confidence interval, *P p* value.

Bold values indicate statistical significance.

All models were adjusted for age, sex, marital status, employment status, frequency of getting together with family, and frequency of getting together with friends

## Data Availability

Data employed in this study is available via request through the Luxembourg Institute of Socio-Economic Research (LISER). https://www.liser.lu/ise/source_of_data.cfm?id=151
